# Selenium as a Protective Agent against Pests: A Review

**DOI:** 10.3390/plants8080262

**Published:** 2019-08-01

**Authors:** Špela Mechora

**Affiliations:** Agency for Radwaste Management, Celovška cesta 182, 1000 Ljubljana, Slovenia; spela.mechora@gmail.com

**Keywords:** selenium, pest, deterrence, toxicity

## Abstract

The aim of the present review is to summarize selenium’s connection to pests. Phytopharmaceuticals for pest control, which increase the pollution in the environment, are still widely used nowadays regardless of their negative characteristics. The use of trace elements, including selenium, can be an alternative method of pest control. Selenium can repel pests, reduce their growth, or cause toxic effects while having a positive effect on the growth of plants. In conclusion, accumulated selenium protects plants against aphids, weevils, cabbage loopers, cabbage root flies, beetles, caterpillars, and crickets due to both deterrence and toxicity.

## 1. Selenium: A Powerful Element

The element selenium (Se) has a narrow range between essentiality and toxicity for living organisms (one order of magnitude). The perception of this element changed over time. It was known as toxic to organisms until its essentiality was discovered [1]. Se is chemically similar to sulphur (S), which leads to nonspecific binding of Se instead of S. This disrupts cell metabolism [2,3] and changes the structure of proteins [4] resulting in toxicity. 

The element is found in the lithosphere, soil, water, and air, but the distribution varies around the world. Deficit of Se can be found on the Russian Plane (0.01 mg kg^−1^) while toxic concentration of 1200 mg kg^−1^ can be found in Meath, Ireland [5]. There are also some places which have Se pollution problems, the source of which can be natural or anthropogenic. 

The essentiality of Se as a nutrient was proven for humans and animals only, while for higher plants it is a beneficial element. However, it is required for optimal growth of green algae *Chlamydomonas reinhardtii* P.A. Dang. [6]. 

## 2. Forms of Selenium and Their Effect

Se has six known isotopes: ^74^Se, ^76^Se, ^77^Se, ^78^Se, ^80^Se, and ^82^Se. In inorganic and organic compounds, the element exists in various oxidative states: elemental Se (0), selenide (−2), selenite (+4), and selenate (+6). In the environment, it can be found in various organic forms; gaseous (dimethylselenide, dimethyldiselenide) and nongaseous (SeCys_2_, SeMet, SeMeSeCys) [7]. The availability and distribution of Se depend on the pH and content of organic matter in soil, redox conditions, competing ionic species such as S, mineralogy, microbial activity, soil texture, temperature, and moisture [8]. Selenate predominates in aerobic soils with neutral pH, whereas selenite prevails at lower pH and redox potential. Selenide predominates under strongly reduced soil conditions [9].

The bioavailability and also the toxicity of Se is determined by its forms [10]. In lower concentrations Se has a positive effect on human health; inhibits some forms of cancer [11], has neuroprotective [12] and immunological effects (selenoproteins regulate immune cell functions) [13] and inhibit the promotion of HIV [14]. Insufficient concentrations of Se in humans can cause hypothyroidism, heart disease, and weaken the immune system [15]. 

Se may also be beneficial for plants. In lower concentrations it acts as an antioxidant [16]; increases plant tolerance to drought [17], salt [18], and UV induced stress [19], and decreases the uptake of toxic metals or reduce their oxidative effect [20]. This can contribute to a greater yield and quality of food, while Se has a positive effect on growth and development of plants [21,22]. High amounts of Se can lead to toxicity in plants which shows as chlorosis, inhibited growth, reduction in protein synthesis, and necrosis [23]. 

Since plants are food for many animals (e.g., cattle), Se deprivation can cause many diseases. Signs of Se deficiencies were reported in a number of species (livestock, pigs, poultry). Short term effects of Se deficiency were found to impair the growth, health and fertility of livestock. Deficiency of Se causes white muscle disease, a myopathy affecting both cardiac and skeletal muscles in young animals [24] and animals often die because of heart failure. The toxic effects of Se on livestock were observed in South Dakota in 19th century, where poisoning could be related to high levels of Se in native plants [25]. Today this is called selenosis, a chronic Se toxic effect [26], which is mostly caused by ingestion of Se accumulators.

## 3. Plant Uptake 

**Figure 1:**
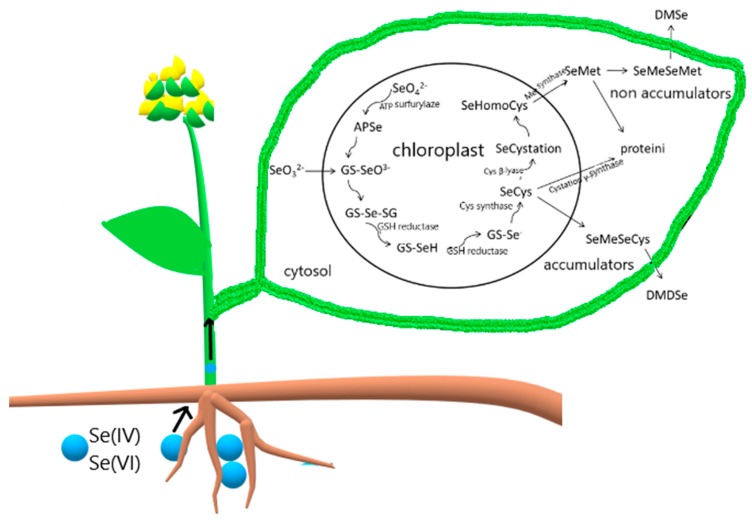
Se uptake and metabolism in the leaves.

There is a strong link between Se concentration in soil and uptake of Se into plants, however the availability of Se from soil is more important than the total Se content in the soil. Inorganic Se species found in soil are selenide, elemental Se, selenite and selenate [7]. Both inorganic forms are highly soluble and more available to plants although selenate is taken up more readily than selenite [27]. Selenate is taken up into the root via sulfate transporters [28] while selenite enters the plants by passive transport. Selenate is distributed from the roots to other parts of the plant via the xylem. Metabolism of inorganic forms to SeCys is located in chloroplasts [29] therefore the selenate transformation takes place in the leaf. Further on SeCys is transformed to SeMet and volatile forms [29] (Figure 1). Organic forms are then translocated via phloem to the roots and other organs [30]. Selenite readily transforms to SeMet which accumulates mainly in the roots. 

The majority of plants thrive in environments with moderate concentrations of Se but can still concentrate it in their tissues. It is known that plants with a lot of sulfur (S) in the tissues do not suffer damage as a consequence of uptake of high amount of Se. Primary accumulators like *Stanleya pinnata* (Pursh) Britton (Brassicaceae) and *Astragalus bisulcatus* (Hook.) A. Gray (Fabaceae) can accumulate beyond 1000 µg Se g^−1^ DW, while *Brassica juncea* (L.) Czern. (Brassicaceae) accumulates less than 1000 µg Se g^−1^ DW and is so called secondary accumulator [31]. Metabolism of Se in accumulators and nonaccumulators differs; nonaccumulators incorporate SeCys and SeMet into proteins, while accumulators these forms transform to SeMeSeMet and SeMeSeCys and further on to volatile forms (Figure 1) [29,32]. An example of such a plant is *Brassica juncea* [33]. Nonaccumulators have a slower rate of assimilation of Se and therefore accumulate more inorganic Se [30,34].

When plants uptake Se from the soil the element enters the food chain. Se can biotransfer through the food chain, from plants, to invertebrates and vertebrates. Possible biotransfer of Se from plants to insects may not have consequences for the insects [35] but for the animals that feed on them [36]. 

The use of accumulators for phytoremediation is already widely used, but the connection between accumulators and pests is poorly studied. The accumulators can clean up the soil of Se and at the same time reduce local populations of a pest (*Spodoptera exigua* on *Atriplex* lines) [37] but because of possible biotransfer of Se more research should be done in the future. Therefore, the accumulators should not support the growth of agriculturally important insects [37]. 

## 4. Connection between Selenium and Pests 

Se has a great impact on insect physiology [38]. Studies showed that insects accumulate Se, but its effect on insect growth and survival is less investigated [35]. Studies have used very high concentrations of Se and focused only on enzymes, but not on the ecological effect of Se toxicity. Trumble et al. [35] demonstrated that terrestrial herbivores suffer high mortality at concentrations corresponding to levels in nonaccumulator plants. 

### 4.1. How Does Selenium Affect Pests? 

**Figure 2:**
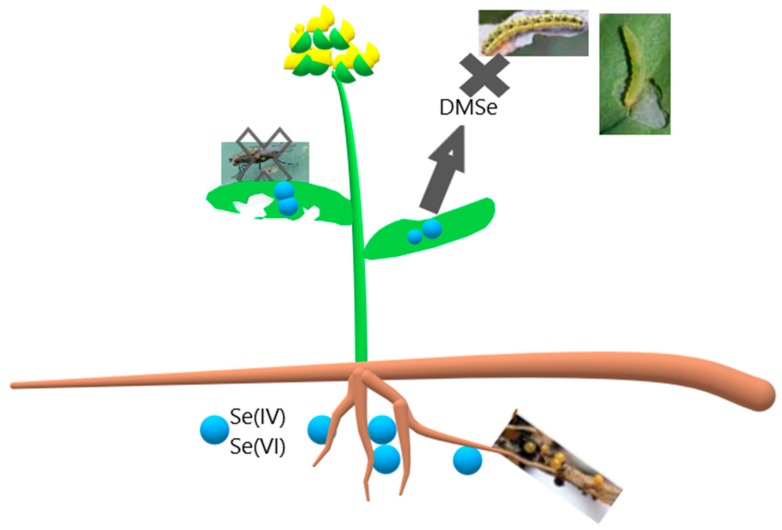
Se forms with deterrent effect on pests. DMSe—dimethylselenide, Se(IV)—selenite, Se(VI)—selenate.

Plant’s chemical protective mechanism against pests can be achieved with the accumulation of elements, including Se, which is explained in the hypothesis of elemental defense [39]. This hypothesis is supported with many studies where the demonstration of elements acting as a deterrent agent against pests was carried out; elements protected the plant against the plant’s feeder, suppressed pest’s growth, impaired their reproduction or caused death (Figure 2). 

At the end of the twentieth century, the attention on Se effects on insects increased. The first study about the toxic effects of Se on insects was performed at the end of 1930s. The earliest studies supported the information about accumulation of Se in insects without consideration of consequences on growth and survival [40,41]. 

Phytoremediation of seleniferous soils triggered more studies focused on a link between accumulator plants and insect pests and the pathway of Se. Se accumulators have a protective mechanism which is based on (1) high (toxic) amount of Se in a plant, (2) Se gas forms deterring the pests (Figure 2) [33], or (3) compounds which start to accumulate in the presence of Se and have a deterrent effect (glucosinolates). 

The amount and Se forms in plants have an impact on the population dynamics of insect herbivores. As mentioned in Section 3, plants uptake Se from the soil and metabolize it to organic forms. In particular, selenate is a deterrent agent against the plant’s feeders, although selenite is more toxic. Organic compounds are less common to avert pests, but gaseous forms released by accumulators (dimethylselenide, dimethyldiselenide) [31] (Figure 2) may be the deterrent compound. 

Direct toxicity and reduction in development together limit the population sizes [35]. The number of mature specimens is affected by toxicity, which also causes delayed development and adds to mortality due to environmental and biotic factors. 

The element accumulates preferentially in the malpighian tubules and in the midgut of insects [40]. After the saturation point has been reached, the content increases in the rest of the body. The excess of Se insects reduce by excretion (malpighian tubules, gland secrets, excretion at each molting, etc.) [42]. 

Like any plant defense, evolution led to the adaptation of some herbivores to selenomethionine which has an environmental implication [35,43]. For example, the diamondback moth (*Plutella xylostella* (Linnaeus)) has the physiological mechanism of detoxification—the methylselenocysteine is accumulated instead of selenocysteine which is accumulated in other related moths, which allows it to grow on accumulators due to adaptation to toxic amounts of Se [44]. 

### 4.2. Spodoptera exigua

Beet armyworm *Spodoptera exigua* (Hübner) (Lepidoptera: Noctuidae) is an economically important pest which is a polyphagous feeder on plants from families Fabaceae, Chenopodiaceae, Solanaceae, Malvaceae, Amaranthaceae, Asteraceae, and Apiaceae. 

Studies in the laboratory showed that a diet enriched with Se acts as antifeedant for larvae of *S. exigua* and influence the selection of plants and feeding site [35,43]. The concentration of 12 µg g^−1^ inorganic Se in the diet reduced pupal weights, while SeMet had no difference [35]. The lethal dose for *S. exigua* differed between inorganic Se forms. The time to complete development of larvae was prolonged for over 25% and the development from egg to adult was prolonged by up to 30% in the presence of Se. Lower concentrations of Se(VI) slowed the growth rate, while the growth rate of the pest dropped by over 90% in the presence of Se(IV). The study showed greater toxic effect of inorganic Se compared to organic ones (selenomethionine), where no effect was observed [35]. 

In another study, only 20% of the larvae were found on the Se diet comparing to a Se free diet. With increasing Se concentrations in the diet even lower percentage of larvae were found on a treated diet then before [43]. Diet with selenite reduced the number of instar larvae by 70–75% [43]. On the other hand, early instars did not avoid the diet with organic Se. Older larvae showed more tolerance then younger, but both selectively avoided a diet with inorganic Se, where there was no antifeedant activity to organic Se (SeMet, SeCys). Inorganic Se proved to be an antifeedant [43]. 

*Medicago sativa* L. treated with high levels of Se(VI) accumulated Se to great extent and consequently the growth, development and survival of *S. exigua* were significantly reduced [45]. In the field, varieties of *Atriplex* lines were treated with Se(VI) and took up high amount of Se which caused mortality of mostly larvae of *S. exigua* and not pupae [37]. Development of insects to the adult stage lasted longer than normal. The reduced population of *S. exigua* on *Atriplex* lines was also observed in another study [46]. 

### 4.3. Trichoplusia ni

Cabbage looper *Trichoplusia ni* (Hübner) (Lepidoptera: Noctuidae) is a polyphagous insect herbivore. The growth of larvae was inhibited by 40% with addition of 25 µg Se(IV) g^−1^ or more in the diet [47]. The growth of larvae *T. ni* was severely affected by higher Se concentrations [47]. The growth of larvae *T. ni*, fed on a Se diet first and later transferred four instar larvae to a Se free diet, was significantly inhibited compared to larvae fed on a Se free diet from the beginning [47]. On the other hand the transferred larvae from a Se free diet to Se diet lagged in growth but by the fifth instar caught up the growth of larvae from Se free diet.

Feeding site preference of a cabbage looper (*T. ni*) was affected by Se [46]. Pupae of the cabbage looper preferred control plants to Se containing plants. The transfer of Se from plant *B. juncea,* containing 465 µg Se g^−1^, to pest *T. ni* was observed which led to higher mortality of the pest. Cabbage loopers that survived, bioaccumulated Se up to 2960 µg Se kg^−1^ in the body [46].

### 4.4. Pieris rapae 

White cabbage butterfly caterpillar *Pieris rapae* (Linnaeus) (Lepidoptera: Pieridae) is a herbivore which feeds only on *Brassica* plants. In the laboratory study, the choice feeding experiment was conducted. Caterpillar larvae chose to feed on leaves without Se, which resulted in lower percentage of leaf damage than the leaf containing Se [33]. Leaves with Se were touched (eaten) which means that caterpillars tested them first. Se severely affected the development of larvae. Newly hatched larvae, placed on plants containing 1.3 mg Se g^−1^, died within 9 days. Older larvae, placed on plants with Se (1.6 mg g^−1^), died after two days. In the first, nine days old larvae lost the weight by 20% and the next day all caterpillars died [33]. 

### 4.5. Myzus persicae

Green peach aphid *Myzus persicae* (Sulzer) (Aphididae) is a general phloem-feeder. 

After green peach aphids invaded the plants of *Brassica juncea*, more were found on Se free plants than plants with added Se and after a week almost all aphids were found on a Se free plants [48]. Aphid colonization was negatively affected on *B. juncea* plants containing 10 mg Se kg^−1^. The lethal dose for aphids was set at 125 mg kg^−1^.

The second part of the experiment by Hanson et al. [48] was to test if Se can act as an antifeedant against aphids. Plants grown hydroponically in a solution containing 0.053 µg Se(VI) L^−1^ or were foliarly sprayed with 0.11 µg Se(VI) L^−1^ dose. Plants in hydroponic system took up greater amount of Se, therefore the toxicity of plants for aphids was high which reduced population of aphids up to 75%. On the other hand, foliar treatment reduced aphid population only by 20% [48].

### 4.6. Acheta domestica

Brown crickets *Acheta domestica* (Linnaeus) (Orthoptera) is a polyphagous herbivore. Crickets were used in a choice feeding experiment [49]. Two accumulators of Se were treated with Se(VI), i.e., *S. pinnata* and *B. juncea*. When given a choice, crickets preferred the plants without Se to plants with Se. In a nonchoice feeding experiment crickets preferred to feed on plants with lower amount of Se (230 µg Se g^−1^) to plants that contained 447 µg Se g^−1^ [49]. The mortality of crickets was greater on the diet with high Se than crickets on a low Se diet. 

### 4.7. Sitophilus granarius

The wheat weevil *Sitophilus granarius* (Linnaeus) (Coleoptera: Curculionidae) is a common pest known worldwide. It can cause significant damage to harvested stored grains and may drastically decrease crop yields. This may be prevented by Se application [50]. Addition of Se(IV) to the soil (90, 270, 810 kg ha^−1^) increased Se content in the triticale grain which resulted in significantly lower percentage of damaged grains because of wheat weevil [50].

### 4.8. Phyllotreta spp. and Delia radicum

Flea beetles *Phyllotreta* spp. (Coleoptera: Chrysomelidae) and cabbage root fly *Delia radicum* (L.) (Diptera: Anthomyiidae) are *Brassica* pests. Adult beetles can be found on the undersides of leaves where they do most of the damage [51]. Cabbage root fly is a major root feeding pest [52]. 

Broccoli transplants treated with 50 mg Se(VI) L^−1^ were slightly less damaged due to *Phyllotreta* spp. [53]. Added Se affected oviposition of first generation of *D. radicum;* 14 and 20 eggs per plant per control and per plant with Se were laid, respectively [53]. The second generation laid 15 eggs per control plant and plant with Se. Plants with Se had less pupae of *D. radicum* at harvest as controls [53]. Se bioaccumulated up to 0.8–3.2 µg Se g^−1^ in the body of pupae [53].

In the pot experiment broccoli plants, treated with 50 mg Se(VI) L^−1^, contained 46 µg g^−1^ [53] which exceeds the limit for safe consumption. For human consumption a concentration around 0.1 mg kg^–1^ represents an appropriate amount of Se in consumed vegetables [54], but it is necessary to know in which form Se is present.

### 4.9. Tenebrio molitor

Mealworm *Tenebrio molitor* (Linnaeus) (Coleoptera: Tenebrionidae) larvae feed on stored grains. In a laboratory study a newly emerged mealworm grown in a solution containing 0.125%, 0.25%, or 0.5% sodium selenite [40]. A pronounced toxic effect of Se was observed, resulted in death of beetles. It was found out that the greatest amount of Se is accumulated in a malpighian tubules, the digestive tract was second and the lowest amount of Se was found in a reproductive tissues [40].

## 5. Conclusions

Selenium is a beneficial for the plants and their growth and when accumulated it can act as a repellant against pests. Inorganic Se forms are more readily absorbed by plants and are further metabolized to organic forms. Se protects plants in two ways: it can accumulate in the tissues to toxic concentrations for feeders or it can be metabolized to gaseous forms which act as a repellent.

Based on all the studies presented, Se may be used as an insecticide therefore could serve as an alternative to phytopharmaceuticals in agricultural plant production. The use of Se and the repellant effect is highly dependable on the application of Se and its concentration. Se application to crops could function as a food fortifier as well as an insecticide, but the practice should be carefully considered due to the possibility of biotranfer in the food web.
